# Trauma, alcohol and drugs misuse in car and motorcycle drivers: a prevalence study in a level one trauma center

**DOI:** 10.1007/s13304-021-01131-2

**Published:** 2021-09-13

**Authors:** Federica Renzi, Elisa Reitano, Davanzo Franca, Osvaldo Chiara, Stefania Cimbanassi

**Affiliations:** 1 General Surgery and Trauma Team, ASST Niguarda, Piazza Ospedale Maggiore 3Milano, 20162 Milan, Italy; 2 Division of General Surgery, Department of Translational Medicine, Maggiore Della Carità Hospital, University of Eastern Piedmont, Corso Giuseppe Mazzini 18, Novara, Italy; 3 Milan Poison Control Centre, ASST Grande Ospedale Metropolitano Niguarda, Piazza dell’Ospedale Maggiore 3, 20162 Milan, Italy; 4 General Surgery and Trauma Team, University of Milan, ASST Niguarda, Piazza Ospedale Maggiore 3Milano, 20162 Milan, Italy

**Keywords:** Trauma, Alcohol, Drugs, Trauma center

## Abstract

Alcohol and drugs misuse represents an important social problem. There is no agreement about influence of ethanol and drugs on trauma severity and clinical course. The aim of this study was to investigate the impact of alcohol and drugs abuse on road related trauma managed to our Level I Trauma Center. Data of 1067 car or motorcycle drivers consecutively admitted in a 5 years period were retrospectively analyzed. The sample was divided into two groups: patients with alcohol and/or drugs misuse and patients without detectable plasmatic levels or not screened because no clinical suspicion of these substance. Demographic data, mechanism of trauma, severity of injury, daily and season time of trauma distribution, alcohol and drugs levels and outcomes were retrieved. Alcohol or drugs misuse were detected in 242 patients. Heavy alcohols levels were the 62.3%. Among drugs cannabis was the most detected substance. These patients were significantly younger than the overall study population (*p* = 0.011), with a higher ISS (*p* = 0.012) a lower RTS (*p* = 0.047), a lower GCS (*p* = 0.005) and an higher head injuries severity (*p* = 0.030). Regarding time distribution, Saturday was the day with the highest percentage of trauma associated with substance misuse (21%). Alcohol/drugs misuse plays a very important role in the epidemiology of road related trauma. Despite the higher severity of trauma scores and the higher incidence of severe head injuries in patients with alcohol or drugs consumption, there were no effects of this substances on mortality of injured patients involved in road crashes.

## Introduction

Road accidents play an important role in public health [1]. According to the latest report of the World Health Organization (WHO) [2], about 1.25 million people die each year as a result of road accidents, and people involved in non-mortal crashes are between 20 and 50 million. Among road accident risk factors, alcohol and illicit drugs use, seems to be increasingly dominant, [3]. As shown by WHO’s report about global status of road safety [2], only 34 countries have drink-driving laws in line with the best practice. Among these countries, 21 are in the European Region.

The interactions of alcohol and drugs on the driving skills are well known and proven. Alcohol affects the neuropsychic functions, interacting with sensory-motor and behavioral functions resulting in alterations of visual perception, reaction times, ability to concentration and judgment [4–6]. The neurotoxic action of drugs leads to stimulation effects, depression, hallucinatory phenomena, and consequent impairment of driving performance [3].

Different epidemiological studies demonstrated that alcohol misuse, reducing driving performance, increased risk of road accidents [7, 8]. However, there is low evidence of the association of drugs abuse and road accidents [3, 9] and literature is conflicting. Different authors agree on the association of alcohol and illicit drugs misuse with an increased need of Intensive Care Unit (ICU) [10–12] and with a higher morbidity, while no associations were found with the Glasgow Coma Scale (GCS) level, hospital length of stay (LOS), mortality rate [11], sepsis or multi-organ failure (MOF), [13]. Different studies demonstrated no associations between the alcohol or drugs misuse and the severity of trauma, estimated with the Abbreviated Injury Scale (AIS) and the Injury Severity Score (ISS) [11, 12, 14]; However, other studies supported the relationship between alcohol/drugs misuse and trauma severity [15–17]. The aim of this study is to provide an epidemiological description of alcohol and drugs use in drivers and to investigate the impact on the injury severity, comparing patients who used one or more substances with patients who did not.

## Methods

All data on car and motorcycle-related trauma consecutively admitted to the Niguarda Trauma Center from January 2011 to December 2015 were retrieved from our Trauma Registry. This study was conducted in conformity to the principles declared to the National Commission for Data Protection and Liberties (CNIL: 2210699) and in accordance with the ethical principles described in the Declaration of Helsinki. Demographic data, mechanism of trauma, type of vehicle, Abbreviated Injury Scale (AIS), Injury Severity Scale (ISS), daily, seasons time and outcome were retrospectively analyzed. The Italian law provides for the possibility of verifying the level of illicit substances in the blood or urine of drivers only (a) at the request of the police or (b) due to the clinical suspicion by the doctor. In the second condition, informed consent should be obtained from conscious patients.

Plasmatic levels cut-off of illicit substances were [18]: ≥ 50 mg/dl of alcohol; ≥ 300 ng/mL of benzodiazepines, ≥ 300 ng/mL of urinary metabolites of cocaine ≥ 300 ng/dL of opiates and ≥ 50 ng/mL of delta-9-tetrahydrocannabinol (THC) (cut-off > 50 ng/mL).

According with toxicological analysis results, patients were identified as:

– Patients who used one or more substances (Group 1);
– Patients with no detected substance or not screened for drugs use (Group 2);
– Patients with high blood levels of opiates and/or benzodiazepines, with documented administration of these substances by pre-hospital health care personnel were included in the Group 2.

In the Emergency Department the trauma leader decided to carry out toxicological exams in subjects with behavioral modifications or altered physical exam or vital signs suggesting alcohol or drugs abuse, such as alcohol smell of breath, ocular signs (miosis), tachycardia, hyper or hypotension, widespread tremors, obtunded consciousness. Sample was further divided in two groups: patients with an ISS lower or greater than 25, in order to assess the trauma severity in accordance to substance misuse. Injuries were grouped by anatomical region: head, chest, abdomen, and extremities according to AIS classification. Five age groups were considered (≤ 17 years, 18–29 years; 30–35 years; 36–50 years and > 50 years) to describe the epidemiological distribution of substance misuse. Finally, differences among car and motorcycle drivers were analyzed to investigate differences on trauma severity and outcome between them.

Data were recorded in a computerized spreadsheet (Microsoft Excel 2016; Microsoft Corporation, Redmond; WA) and analyzed with statistical software (IBM Corp., released 2012, IBM SPSS Statistics for Windows, Version 21.0; Armonk, NY, IBM Corp.). Kruskal–Wallis test was used to compare continue variables while categorical variables were compared using Pearson’s Chi-squared test. Two different multivariate models were performed: the first to identify drugs or alcohol correlation with the injury’s severity and patient’s demographic data; the second to identify any possible correlation between the injury severity and the type of vehicle (car vs motorcycle drivers). Variables with a *P* value below 0.05 at the bivariate analysis were considered statistically significant and were included in the logistic regression model.

## Results

During the study period, 2811 trauma patients involved in road traffic related accidents, were admitted to our Trauma Center. Car and motorcycle drivers who fulfilled the inclusion criteria were 1067. The mean age of the study population was 44 years (10–92 years) and 925 (86.7%) were male. The overall mortality was 3.3%.

Subjects with positive toxicological analyses (group 1) were 242 (car drivers 91, 37.6%; motorcycle drivers 151, 62.4%). Group 2 patients were 825: 164 (31.7%) car drivers and 354 (68.3%) motorcycle drivers.

Patients with heavy alcohol consumption accounted for 62.3%. Cannabis was the most detected drug (39.3%), followed by cocaine (27.3%), opiates (1.7%) and benzodiazepines (1.2%).

Motorcycle drivers were found to be more frequently positive for drugs misuse than car drivers (*p* = 0.104). General characteristics of the two groups are resumed in Table 1. Logistic regression model showed no correlation between drugs/alcohol misuse and trauma severity with the exception of a higher injury severity (Head AIS) in Group1.

**Table 1:** Demographic data between the groups

	Group 1 242	Group 2 825	*P* value
Male *n* (%)	219 (90.5)	706 (85.6)	0.048*
Age median (IQR)	42 (33.75–51)	45 (34.50–55.0)	0.011*
Car drivers *n* (%)	91 (37.6)	264 (32)	0.104
Motorcycle drivers *n* (%)	151 (62.4)	561 (68)	0.104
GCS median (IQR)	15 (14–15)	15(15–15)	0.005*
RTS median (IQR)	12(11–12)	12 (12–12)	0.047*
ISS median (IQR)	11 (5–21)	9 (4–21)	0.012*
Death probability median (IQR)	0.80 (0.4–3.0)	0.60 (0.40–3.0)	0.096
Dead *n* (%)	7 (2.9)	28 (3.4)	0.700
Head AIS ≥ 3 *n* (%)	57 (38)	140 (28.6)	0.030*
Chest AIS ≥ 3 *n* (%)	82 (83.7)	247 (81.5)	0.629
Abdomen AIS ≥ 3 *n* (%)	23 (43.4)	78 (42.2)	0.873
Extremity AIS ≥ 3 *n* (%)	51 (38.3)	163 (37.6)	0.870

*GCS* Glasgow Coma Scale, *RTS* Revised Trauma Score, *ISS* Injury Severity Score, *AIS* Abbreviated Injury Scale

*Statistical significance

Sixty-two patients of group 1 (25.6%) were found to be positive for the contemporary use of different substances (Multi-drugs association-MDA). Between the MDA, the association cocaine-alcohol was the most detected (32 patients, 13.2%), followed by the association cannabis-alcohol (31 patients, 12.8%). However, patients were often positive for more MDA: Cocaine-alcohol-cannabis association was the most detected (14 patients, 5.8%). Analyzing the MDA in relation to the type of vehicle, MDA was more associated with motorcycle drivers (*p* = 0–041).

Patients with substance abuse were significantly younger than the overall study population (42 years, IQR 33.75–51.00 vs 45 years, IQR 34.50–55; *p* = 0.011), with an higher ISS (11, IQR 5–21 vs 9 IQR 4–21; *p* = 0.012) and an higher although not significant death probability (0.80 IQR 0.40–3.0 vs 0.60, IQR 0.40–3.0; *p* = 0.096). Moreover group 1 patients showed a lower GCS (*p* = 0.005) and RTS (*p* = 0.047). Injuries with head AIS ≥ 3 were found to be more frequent in group 1 (*p* = 0.030), while no difference was found between the AIS of the remaining body districts (Table 1).

Injuries with AIS ≥ 3 in at least one body region were found in 544 (51.0%) of which 138 patients in group 1 (57%): 94 motorcycle drivers (62.3%) and 44 car drivers (48.4%), *p* = 0.034. In group 2 injuries with AIS ≥ 3 in at least one body region were found in 406 patients (49.2%): 308 motorcycle drivers (54.9%) and 98 car drivers (37.1%), *p* ≤ 0.001. A significant overall difference was found between the two groups (*p* = 0.03).

**Fig. 1:**
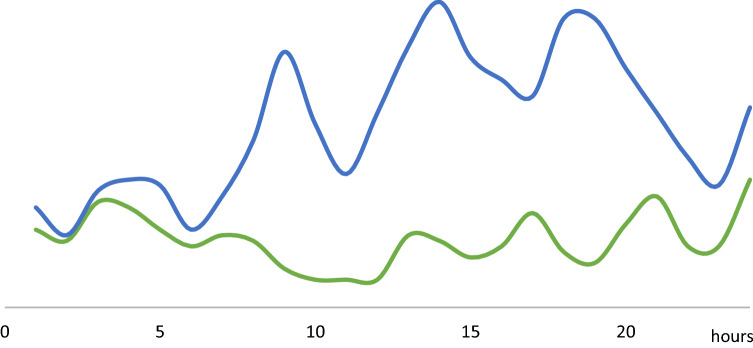
Daily distribution of substance misuse in trauma (Green line: GROUP 1; Blue line: GROUP 1 and 2)

In group 1 patients after a peak in the early hours of the day (00AM–06AM) the number of crashes decreases, then increasing again, with a variable progression after noon (Fig. 1). Alcohol and drugs misuse was most frequent on Saturday (21%), followed by Sunday (18.3%) and Thursday (15.9%).

Thirty-five patients died: 15 car drivers and 20 motorcycle drivers. They were mainly male (94.3%) and significantly older than survived patients (median 55 years, IQR 39–63 vs. 44 years, IQR 34–54; *p* = 0.004). Seven patients among them were found to be positive at toxicological analyses. Dead patients showed a lower GCS (median 3, IQR 3–3 vs median 15, IQR 15–15; *p* ≤ 0.001), a higher ISS (median 45, IQR 37–75 vs median 9, IQR 5–19; *p* ≤ 0.001) and a higher RTS (median 2, IQR 2–4 vs median 12, IQR 12–12, *p* ≤ 0.001) if compared with survived patients. The rate of deaths was not significantly different between group 1 and 2 (Table 1). There was no difference for positivity of alcohol or drugs misuse in patients with an ISS lower or greater than 25 (Table 2). In Table 3 the distribution of substance misuse in different age groups is described: differences between the age groups were statistically significant for alcohol and cannabis misuse, with the age group between 36 and 50 years old more often positive at the screening tests. This age group was found to be more often positive also for cocaine, opiates and benzodiazepine misuse, even if no statistical difference between the other age groups was showed. Table 4 showed comparison among car and motorcycle drivers: no differences were showed in terms of drugs misuse between the groups. Multivariate logistic regression showed no differences in terms of injury severity in the different body areas, while a positive correlation was found between the ISS level and the motorcycle drivers.

**Table 2:** ISS in patients with alcohol or drugs misuse

Substances	Patients positive for substance misuse	ISS < 25	ISS ≥ 25	*P* value
Ethanol *n* (%)	151 (14.2)	121 (80.1)	30 (19.9)	0.732
Cocaine *n* (%)	66 (6.2)	47 (71.2)	19 (28.8)	0.105
Opiates *n* (%)	4 (0.4)	3 (75)	1 (25%)	0.841
Cannabis *n* (%)	95 (8.9)	7 (80)	19 (20)	0.817
Benzodiazepine *n* (%)	3 (0.3)	1 (33.3)	2 (66.7%)	0.051

*ISS Injury Severity Score

**Table 3:** Substance misuse by age groups

Substances	≤ 17 years	18–29 years	30–35 years	36–50 years	≥ 50 years	*P* value
Ethanol *n* (%)	0	10 (6.6)	35 (23.2)	65 (43)	41 (27.2)	0.003*
Cocaine *n* (%)	0	3 (4.5)	17 (25.8)	26 (39.4)	20 (30.3)	0.103
Opiates *n* (%)	0	0	1 (25)	2 (50)	1 (25)	0.905
Cannabis *n* (%)	0	9 (9.5)	27(28.4)	39 (41.1)	20 (21.1)	0.002*
Benzodiazepine *n* (%)	0	0	0	1 (33.3)	2 (66.7)	0.809

*Statistical significance

**Table 4:** Differences between car and motorcycle drivers

	Car drivers 355	Motorcycle drivers 712	*P* value	*P* value Logistic regression (95% CI)
Male *n* (%)	258 (24.2)	667 (62.5)	≤ 0.001*	≤ 0.001* (5.71–71.28)
Age median (IQR)	43 (34–56)	44 (34.25–54)	0.728	
Drugs/alcohol abuse *n* (%)	91 (37.6)	151 (62.4)	0.104	
GCS median (IQR)	15 (15–15)	15(15–15)	0.625	
RTS median (IQR)	12 (12–12)	12 (12–12)	0.140	
ISS median (IQR)	5.5 (2–17)	10 (5–22)	≤ 0.001*	0.661 (0.97–1-03)
Death probability median (IQR)	0.60 (0.3–2.5)	0.80 (0.40–3.0)	≤ 0.001*	0.029* (0.97–0.99)
Dead *n* (%)	15 (1.4)	20 (1.9)	0.221	
Head AIS ≥ 3 *n* (%)	55 (8.6)	142 (22.2)	0.021*	0.054 (0.98–3.97)
Chest AIS ≥ 3 *n* (%)	97 (24.2)	232 (57.9)	0.044*	0.182 (0.78–3.54)
Abdomen AIS ≥ 3 *n* (%)	32 (13.4)	69 (29)	0.866	
Extremity AIS ≥ 3 *n* (%)	46 (8.1)	168 (29.6)	0.867	

*GCS* Glasgow Coma Scale, *RTS* Revised Trauma Score, *ISS* Injury Severity Score, *AIS* Abbreviated Injury Scale, *CI* confidence interval

*Statistical significance

## Discussion

Alcohol and/or drug misuse plays a very important role in the epidemiology of road related trauma. The effects of these substances on neuropsychic and motor functions result in a reduction of driving performance, increasing the risk of accidents [19–21]. A detailed profile of these trauma patients is complex to draw, representing an extremely heterogeneous population with different involved variables, such as [22, 23]:

- Psycho-physical features of patients (age, comorbidities, metabolism);
- Environment, socio-cultural habits and lifestyle influencing accessibility and availability of drugs and affecting the type of abuse and the frequency of assumption (dependence VS occasional consumers);
- Road safety (road conditions, visibility), respect of the Road Code and vehicle type (use of seat belts, car equipped with airbags).

In our study, the 22.68% of the drivers of vehicles showed to be positive for alcohol or drugs misuse, despite the drink and drugs-driving laws.

Different authors [24, 25] investigated the epidemiological distribution by age on alcohol and drugs misuse on road traffic injuries in other countries. In line with these results, our analysis showed that drugs and alcohol misuse are more frequent in male of middle age (36–50 years). The highest number of crashes occurred at night and early in the morning, mostly on Saturday and Sunday, classically associated with increased social events and moments of aggregation. The high frequency of road accidents on Thursdays could be related to the latest social habits in Milan. In the last years, in fact, there was an increase in social events right on this day of the week. Demographic features of patients in Group 1, as well as the temporal distribution of road crashes, could be related with binge drinking [8, 26, 27]. This phenomenon is characterized by the assumption of a high amount of alcohol in a short time, mainly in the weekend or during parties.

Alcohol was the most common misuse substance (62.4%), regardless of age, sex, and type of vehicle used. Among drugs, cannabis was the most common (39.3%). This widespread use of alcohol and cannabis was likely to be explained in their greater affordability and easy of purchase compared to other drugs, enabling more extensive use not only among young people, who reasonably have more limited economic availability, but also in the middle age.

According to the literature [11, 14, 22], the comparison between group 1 and group 2 showed differences on the severity of the injuries. Patients in group 1 were younger, with a higher ISS, a lower GCS and RTS and a higher Head AIS. However, logistic regression model showed no correlation between drugs/alcohol misuse and trauma severity, with the exception of head injuries (Table 1).

In car drivers’ group, safety devices such as seat belts and airbags probably prevented the direct impact of the chest on steering wheel, dashboard and windscreen. Therefore, the lower GCS on group 1 could be partially due to the sedative effects of drugs or alcohol misuses, but also imputable to an increased trauma severity as a result of substance consumption.

Despite the higher recurrence of at least one AIS ≥ 3 injury in each body regions in group 1, no correlation between ISS and substance misuse was observed (Table 2).

Multi-drugs abuse (MDA) was documented in 25.6% of patients of group 1, mostly motorcyclists. In contrast with literature, the most common MDA association was alcohol and cocaine, instead of alcohol and cannabis [28]. The concomitant use of these two substances is proved to be dangerous, as their mixture leads to the formation of Coca-ethylene (CE); an active metabolite that acts on the central nervous system, increasing dopaminergic activity, with euphoric effect [29]. CE is slowly metabolized by the cerebral cortex and this prolongs the duration of the effects [10]. CE activity is burdened by an important cardiotoxic action leading to arrhythmias, electrocardiographic alterations, reduced cardiac contractility, increased risk of heart disease and increased blood pressure [29, 30]. CE plays a key role during the evaluation of patient hemodynamic, because it could be responsible of some cardio-circulatory alterations that cannot be explained by the injuries found after crash [10].

According to literature [11], there was no significant effect on mortality of alcohol and/or drugs on injured patients involved in road crashes. Our study suggested, however, an overall higher severity in patients with drug misuse, as demonstrated by the higher ISS and the lower GCS and RTS of group 1, although logistic regression model showed only a positive correlation with a higher Head AIS. Indeed, epidemiologic limit of this analysis was the lack of information on drugs abuse in patients directly dead on the scene (not evaluated in a hospital). Different studies [31, 32], based on toxicological analysis performed on the drivers dead on the road evidenced that alcohol was the most commonly detected substance. Therefore, further studies are needed to confirm the impact of alcohol and drugs on mortality. As showed in Table 4, no differences were found in terms of drugs and alcohol misuse between car and motorcycle drivers. However, logistic regression model showed a higher death probability in motorcycle drivers, although no differences were found in terms of mortality between the groups.

The WHO Global Status Report on Road Safety 2015 [2], highlights that in Italy road traffic death involving alcohol were about 25%. This was a high percentage if compared with other European countries (Germany Austria, Ireland, Switzerland, Denmark, Czech Republic, Russia) and no European countries such as China and Brazil where alcohol-related deaths were less than 10%. Worst results were reported in France (29%), Portugal (31%), Australia (30%), New Zealand (31%), Canada (34%) and United States (31%, with higher legal limits: 0.8 mg/dL).

According to WHO Global Status Report on Road Safety 2018 [33], Italy is one of the countries with an overall best practice for drink-driving laws. However, our study showed how current social measures should be improved to avoid road related trauma linked to alcohol and drugs misuse.

This study presented several limitations. Not all drivers involved in road crashes and managed by our Trauma Team were subjected to toxicological analysis. As previously described, the decision to perform these exams was carried out by the Trauma Leader in relation to a clinical suspect. Toxicological analyses performed in all patients could lead to more objective data. However, only few patients with normal vital signs, mostly affected by minor trauma, were not tested because any abuse of prohibited substance was unlikely.

An additional limitation applies the type of analysis performed for drugs. The evaluation of urinary metabolites of cocaine, opiates and cannabis does not provide information about a recent abuse of these drugs, but only a previous use. These metabolites have different elimination times: 15–30 days for cannabis, 2–4 days for cocaine and opiates [18]. The elimination rate depends by a lot of factors such as the metabolism and hydration status of each patient, the tolerance to the substance of abuse, the frequency of use and the amount of drug dose [29]. Having plasmatic levels of these drugs, as for alcohol and benzodiazepines, even if more expensive, would provide more reliable results, both for clinical practice and for prospective studies.

There are, moreover, non-investigated but widely used psychoactive substances, such as ecstasy or MDMA (3,4-methylenedioxy-methane-methane) and “Smart Drugs”. The latter category of substances consists of natural and/or synthetic substances that promote the release of neurotransmitters and act as inducers of neuronal growth, improving cognitive capabilities and concentration. Alongside these “positive effects”, negative effects such as induction of addiction, hallucinations, seizures and psychosis are reported, [34]. The main feature of these products is to be subject to free sale because they have not yet been legally recognized as illicit drugs.

In conclusion, in this study alcohol was the most widespread substance, however, the use of drugs among drivers involved in road accidents was far from negligible and often associated with concomitant alcohol intake, thus resulting in a synergistic effect of these illicit substances. These patients showed an overall higher severity and a positive correlation with a higher head AIS, but no single substance was found to be associated with the cluster of high severity (ISS ≥ 25) injuries. Finally, alcohol and drugs misuse represent an important social problem. Clarify the epidemiological distribution of this phenomena could be crucial to enhancing prevention measures and reducing the related morbidity, with a social cost benefit.

Further epidemiological studies are needed to expand the study sample, possibly considering other hemodynamic and metabolic parameters in order to obtain more complete information.
